# The Unintended Consequences of Telehealth in Australia: Critical Interpretive Synthesis

**DOI:** 10.2196/57848

**Published:** 2024-08-27

**Authors:** Sagda Osman, Kate Churruca, Louise A Ellis, Dan Luo, Jeffrey Braithwaite

**Affiliations:** 1 Centre for Healthcare Resilience and Implementation Science Australian Institute of Health Innovation Macquarie University North Ryde Australia; 2 The Daffodil Centre Sydney Australia

**Keywords:** telehealth, telemedicine, unintended consequences, digital health, eHealth, critical interpretive synthesis, review methodology, literature review, Australia

## Abstract

**Background:**

Despite more than 2 decades of telehealth use in Australia and the rapid uptake during the COVID-19 pandemic, little is known about its unintended consequences beyond its planned and intended outcomes.

**Objective:**

The aim of this review was to synthesize evidence on the unintended consequences of telehealth use in Australia to clarify its impact beyond its planned and intended outcomes.

**Methods:**

We conducted a search of 4 electronic databases: Ovid MEDLINE, Ovid Embase, EBSCO CINAHL, and Scopus. A critical interpretive synthesis approach was adopted for its flexibility and interpretive nature. We extracted data about study characteristics and the types and models of telehealth services. The extracted unintended consequences were coded and mapped into the domains and dimensions of the Australian Health Performance Framework.

**Results:**

Of the 4241 records identified by the search, 94 (2.22%) studies were eligible for data extraction and analysis. Of these 94 studies, 23 (24%) reported largely positive unintended consequences of telehealth associated with health status, while 6 (6%) noted a potential negative impact of telehealth on socioeconomic status. The findings of 4 (4%) of the 94 studies highlighted societal and financial consequences of telehealth beyond the health system. Almost all studies (93/94, 99%) reported unintended consequences under the 5 dimensions of the Australian Health Performance Framework.

**Conclusions:**

Our synthesis offers a framework for understanding the unintended consequences of the use of telehealth as an alternative to in-person care in Australia. While we have documented many unintended benefits of telehealth use, our findings also shed light on many challenges of delivering care via telehealth across different domains and dimensions. These findings hold significant practice and policy-making implications for ensuring safe and high-quality care delivery via telehealth.

## Introduction

### Background

The COVID-19 pandemic has resulted in a rapid increase in telehealth use globally. During the pandemic, telehealth emerged as a promising model of care to improve health care delivery and increase access for underserved communities. In Australia, during the first year of the pandemic in 2020, telehealth use grew almost exponentially because many health services rapidly moved to provide their services online [1-4]. Throughout the pandemic and beyond, a high consumer preference has been reported for telehealth as an alternative to standard care when it is clinically appropriate and safe, with several reported benefits such as flexibility, convenience, and time and cost savings [5-9]. This pattern of a dramatic increase in the uptake of telehealth during the COVID-19 pandemic and, subsequently, its sustained use over time is similar across many other countries, such as the United States, the United Kingdom, and New Zealand [10,11].

Telehealth is broadly defined as using information and communication technologies to provide health care at a distance [12]. This definition includes models where a physician-patient interaction, whether synchronous or asynchronous, is not necessarily required, such as in patient monitoring, online support groups, or consumer mobile apps [12]. However, in this review, we focus primarily on synchronous telehealth or synchronous telehealth in conjunction with asynchronous models that act as an alternative to standard in-person care; this includes one-to-one patient-clinician interactions and models where clinician-clinician interactions appertain to case management.

Unintended consequences are the unplanned and often unforeseen outcomes of introducing an intervention, such as telehealth [13,14]. These outcomes can be (1) positive outcomes (accidental benefits), (2) adverse outcomes (drawbacks), or (3) neutral outcomes with no intrinsic positive or negative value [13,15]. To provide safe and effective care via telehealth, an understanding of its unintended or unexpected consequences is warranted to mitigate drawbacks, optimize unexpected benefits, and understand system-wide and long-term impact. There has thus far been limited research investigating this issue, with only 1 secondary analysis carried out in Canada [14]. Guided by the model proposed by Bloomrosen et al [16] for investigating the unintended consequences of health information technologies, the study by Alami et al [14] documented findings on the unintended consequences of telehealth across various domains. However, the Canadian data sources were primarily evaluation documents that did not account for published empirical research beyond the 10 projects investigated. Furthermore, despite defining unintended consequences as positive, neutral, or negative unforeseen outcomes, the reported consequences were primarily negative, such as rigidity of technology to accommodate existing workflows and processes, cognitive overload, invasion of patients’ privacy, increase in administrative workload, and the additional costs associated with telehealth. Few neutral consequences were documented in the study, such as the restructuring of hierarchical relationships and the emergence of new modes of clinical practice [14].

### Objectives

A comprehensive synthesis of primary and secondary empirical studies on this subject is thus still lacking. In this review, we aimed to close this knowledge gap by synthesizing findings on the unintended consequences of telehealth to fully understand the impact of telehealth, focusing not only on the microlevel (individual users) and the mesolevel (organizational processes and workflows) but also on a policy macrolevel or national level. The objective of the review was to investigate the current state of knowledge on the multilevel unintended consequences of telehealth implementation in the Australian health system. We used a critical interpretive synthesis (CIS) [17] approach due to the diversity of telehealth literature and the lack of consensus on terms and concepts. This review is part of a larger research project using systems sciences as a conceptual lens to understand telehealth’s unintended consequences [13]. This conceptual framework informed the design and analysis of this review, emphasizing the rippling effects of telehealth implementation across the complex health care system.

### Research Questions

The following research questions (RQs) were formulated to achieve the review objective:

- RQ1: How is telehealth implementation being evaluated or assessed?
- RQ2: What are the findings on the types and models of telehealth services implemented?
- RQ3: What are the findings on the unintended consequences of telehealth implementation?

While the first 2 RQs were used to extract study characteristics, the third RQ served as an initial guide for the CIS. In line with the principles of this methodology, our inquiry evolved and was refined throughout the review process, as detailed in the Methods section.

We focused our review on studies conducted within Australia to provide a picture of unintended consequences within a single, contained health care system. Health systems vary across different countries in terms of structure, funding models, and policies, but Australia represents a high-income country with a well-resourced hybrid health system that deals with fragmentation and challenges similar to those in comparable international health systems [18], making our findings relevant to such systems.

## Methods

### The CIS Method

A CIS offers an appropriate alternative to a standard systematic review. Its exploratory nature allows for flexibility in including diverse study types [17], including studies that do not focus directly on the unintended consequences of telehealth or ostensibly on the topic of unintended consequences but could still inform the analysis. Moreover, CIS provided us with the interpretive freedom necessary to build a framework and draw conclusions from studies that reported on the unintended consequences of telehealth without necessarily using the term “unintended consequences.” This interpretive flexibility is necessary when building a knowledge base and synthesizing evidence where the literature is diverse and complex [17].

### Search Strategy

A search strategy was developed in consultation with a clinical librarian to search the databases Ovid MEDLINE, Embase, EBSCO CINAHL, and Scopus using keywords and Medical Subject Headings terms related to “telehealth,” its “implementation,” and “unintended consequences” in “Australia” (Multimedia Appendix 1). We did not apply date limits to the search to ensure a comprehensive account of the available evidence. The search was conducted in November 2022.

### Eligibility Criteria

Qualitative and mixed methods allow for the exploration of issues and variables that might be unexpected and therefore remain unmeasured in quantitative studies. They provide richness and depth, facilitating a better understanding of any unintended consequences of telehealth beyond the planned outcomes [19]; as such, they served as our primary data sources. The inclusion and exclusion criteria are presented in Textbox 1.

**Inclusion and exclusion criteria.**

**Inclusion criteria**

- Published in a peer-reviewed journal
- Examined synchronous telehealth (telephone or video) or synchronous telehealth in conjunction with asynchronous models for service delivery as part of the Australian health system
- Qualitative or mixed methods evaluations that examined the implementation of synchronous telehealth or synchronous telehealth in conjunction with asynchronous models or its unintended consequences in both routine practice or evaluation of specific programs or quantitative studies in which the main focus was the unintended outcomes of telehealth
- Unlimited in setting or specialty because the goal was to identify the unintended consequences of synchronous telehealth as a modality and an alternative model of care regardless of the specialty or setting; however, we extracted setting and specialty data for each study included
- Published in English and available in full text

**Exclusion criteria**

- Reviews, protocols, opinion pieces, commentaries, perspectives, conference proceedings, and books
- Focused solely on asynchronous telehealth modalities: store and forward, patient monitoring, mobile apps, studies on telehealth for education or professional development purposes only, or studies assessing telehealth readiness
- Carried out across multiple countries
- Not available in full text or not in English
- Examining after-hours helpline and triage programs (these were excluded because such programs primarily serve as entry points for assessment and referral services rather than as replacements for standard care [20,21])

### Study Screening

To facilitate screening and duplicate removal, all references were downloaded to a reference manager (EndNote 20; Clarivate) and then uploaded to the web-based screening platform Rayyan (Rayyan Systems Inc). The first author (SO) removed all duplicates, and then 2 reviewers (SO and KC) independently completed a blind review of a random sample of titles and abstracts (2%) against the aforementioned inclusion and exclusion criteria. Cohen κ was calculated (0.82; near-perfect agreement) to determine interrater reliability, and the few discrepancies in the inclusion or exclusion decisions were discussed between the 2 reviewers (SO and KC). When necessary, a third reviewer (LAE) was consulted until a consensus was reached. A single reviewer (SO) screened the remainder of the titles and abstracts against the inclusion and exclusion criteria and conducted the full-text screening of the included references. As there is no search protocol for conducting a CIS, we followed the PRISMA (Preferred Reporting Items for Systematic Reviews and Meta-Analyses) guidelines.

### Data Extraction

Study characteristics and findings on the types and models of telehealth services were extracted into a custom template in NVivo 20 (Lumivero) and Excel (Microsoft Corp). We extracted theoretical frameworks or models only if they underpinned the design and analysis of the study, with sufficient detail provided to explain their application. Textbox 2 summarizes the data items captured to answer RQs 1 and 2. To answer RQ 3, we identified any unintended benefits or drawbacks by examining the reported results in each included study, with the relevant text extracted verbatim and added to NVivo for coding and analysis.

**Data items extracted.**

**Research questions and data items**

- How is telehealth implementation being evaluated or assessed?
  Study design and methods used
  Data collection tools
  Theories and frameworks used to understand the impact of telehealth
  Locations and regions of studies
  Types and number of participants
- What are the findings on the types and models of telehealth services implemented?
  The types of telehealth services implemented
  Settings and specialties
  Models of care implemented
  Whether it was synchronous or a mix of synchronous and asynchronous models
  Modalities of telehealth (video vs telephone)
  If the study was conducted before or after the COVID-19 pandemic
- What are the findings on the unintended consequences of telehealth?
  Any identified unintended consequences
  Whether they are positive, negative, or neutral

Dixon-Woods et al [17] point out that the precise definitions of many constructs in a CIS may evolve during the review process. Our research process exemplified this approach because we began data extraction without a rigid definition of unintended consequences. Instead, we initially extracted all benefits and drawbacks of telehealth from included studies; for instance, we initially considered *reduced travel time for patients* an unintended positive consequence. However, in all included studies, the intended goal of telehealth implementation, whether explicit or implied, was to improve or sustain access to health care services. This definition encompassed using telehealth to ensure continuity of access during the COVID-19 pandemic and to improve access for patients with barriers such as geographic and mobility disadvantages. Therefore, we conducted another round of analysis to refine our understanding of unintended consequences, excluding benefits related to the reduced burden of accessing care in terms of money and time because these were apparently intended outcomes from the studies. Hence, in all instances, an unintended consequence was subsequently defined as either (1) any benefit of telehealth implementation other than improving or sustaining patients’ access to health care services or (2) any drawback because drawbacks are usually not planned or intended although they can be foreseen or expected.

While we recorded whether each study was conducted before or after the COVID-19 pandemic, our data extraction focused on unintended consequences that were not explicitly tied to the unique circumstances of the pandemic. Therefore, we did not extract or include transient consequences solely attributable to the COVID-19 context (eg, consequences related to extreme social distancing measures). Our rationale was that these transient consequences might have limited applicability to long-term, routine telehealth implementation in typical care provision. This methodological approach ensured that our analysis focused on enduring issues relevant to long-term, routine telehealth implementation, regardless of whether the study was conducted before or during the COVID-19 pandemic. Nevertheless, by documenting the temporal context (before or after the pandemic) for each study in Multimedia Appendix 2 [6-8,22-112], we provide the necessary information to interpret the results in light of the implementation context.

### Quality Appraisal

We wanted to be inclusive in our review and not omit any study based on quality issues; therefore, we purposely did not use a structured tool to appraise studies. This is in agreement with the growing case against the exclusion of relevant qualitative studies in reviews on quality grounds alone rather than relevance because they can nevertheless inform and contribute to the understanding as well as the richness of the final findings of a review [17,113,114].

### Data Synthesis and Analysis

To analyze the extracted data, an iterative coding process was used, switching between inductive and deductive coding and following a best-fit framework-based synthesis of evidence [115-118]. This approach allowed us to identify relevant frameworks for analysis as we coded, while minimizing the risk of missing some relevant evidence that did not fit an a priori framework, thus capturing all unintended consequences extracted from the included studies. As we undertook this process, our initial broad question “What are the unintended consequences of telehealth?” evolved into “How do unintended consequences of telehealth manifest across different domains of health care performance?” because it became apparent that telehealth’s unintended consequences could be conceptualized in terms of accessibility, appropriateness, safety, efficiency, effectiveness, and continuity of care. We recognized that these concepts aligned closely with the definitions in the Australian Health Performance Framework (AHPF) [119], a framework with which we were familiar from previous work in the Australian health care context, which is described in detail in the following subsection. We chose to use the AHPF domains instead of defining entirely new constructs as typical when conducting a CIS for the following reasons: (1) the AHPF provided a robust, established framework that could accommodate the complexity of our findings; (2) its use allows for better comparison with other telehealth studies in the Australian context; and (3) it offers a more accessible account of the evidence for policy makers and practitioners.

**Figure 1:**
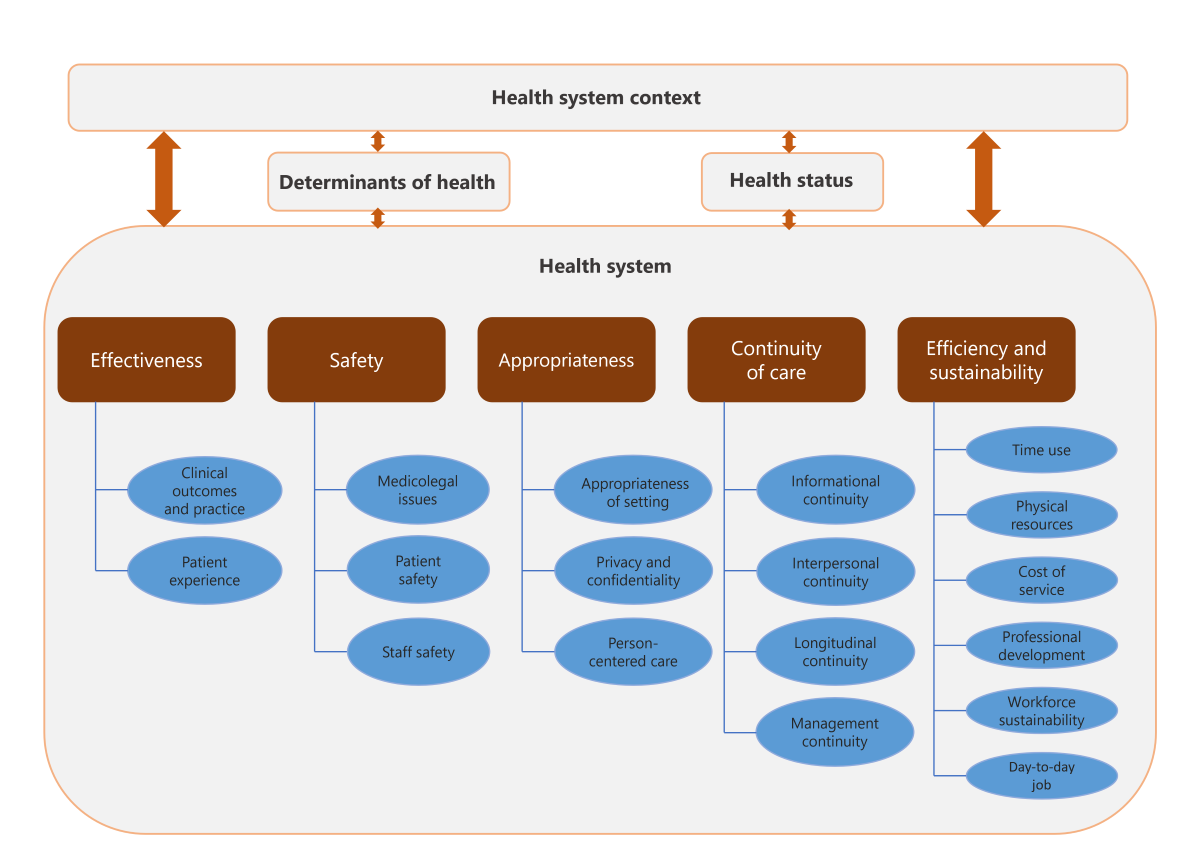
Domains (in gray), dimensions (in brown), and subcategories (in blue) of the extracted consequences mapped into the domains and dimensions of the Australian Health Performance Framework.

Our initial inductive coding process resulted in 137 codes; after an iterative process of refining, merging, and deleting redundant codes, 82 (59.9%) unique codes were deductively mapped and translated into the domains and dimensions of the AHPF. As an example to demonstrate this iterative inductive-deductive process, we initially coded a wide range of positive and negative effects of telehealth related to continuity of care, such as *facilitating multidisciplinary care* and *undermining rapport building*. As we analyzed the data, we recognized that this theme aligned with the *continuity of care* dimension in the AHPF. However, we found it necessary to modify the framework to better summarize and capture consistent variations and nuances in the findings within each dimension (Figure 1); for example, we added subcategories under *continuity of care*, such as *interpersonal continuity* and *management continuity*, to capture telehealth’s unintended consequences related to the *patient-clinician relationship* and *care management across teams*. Thus, we adapted the existing AHPF to document the full impact of telehealth and its unintended consequences within the Australian health system. This approach aligns with the view of Dixon-Woods et al [17] of CIS as a flexible approach that can be adapted to the needs of the RQ, rather than a rigid, prescriptive methodology. NVivo (version 20; Lumivero LLC) was used to facilitate coding and analysis.

### Mapping the Findings to the AHPF Domains

The AHPF supports reporting by different health organizations at different levels to assess the performance of health care delivery in Australia, focusing on four key domains (in gray in Figure 1): (1) determinants of health, (2) health status, (3) health system, and (4) health system context [119]. Within each domain, there are dimensions to guide the assessment of that specific domain; for example, in the health system domain, the AHPF uses accessibility, effectiveness, efficiency and sustainability, safety, appropriateness, and continuity of care as dimensions to guide the assessment of the performance of the Australian health system.

We mapped all extracted consequences into the 4 key domains of the AHPF. Due to the greater abundance of findings falling under the health system domain than under other domains, the system-level consequences constituted most of the evidence presented in this review and necessitated the use of both dimensions and subcategories (shown in brown and blue in Figure 1, respectively), as discussed previously. To map these extracted consequences to the health system domain of the AHPF, we primarily used the definitions provided in the framework for each dimension. Where there was no clear definition, we supplemented with definitions from other sources, as summarized in Textbox 3. While we used the AHPF as an organizing framework, our approach went beyond simple mapping to achieve synthesis. We integrated findings across the included studies to generate new insights into how unintended consequences manifest in telehealth implementation; for instance, by synthesizing the findings related to effectiveness and appropriateness, we developed a new understanding of how telehealth can improve health outcomes by facilitating the involvement of carers in care delivery. By situating our findings within the AHPF, we emphasize telehealth’s overarching impact across various health care performance domains, offering a systems-level perspective on telehealth’s unintended consequences.

**Definitions of the Australian Health Performance Framework health system dimensions as used in data analysis.**

**Definitions and dimensions**

- Accessibility is the ability to access affordable, convenient care at the right time and place, taking into account the various needs and circumstances of patients [119,120]; benefits in terms of time, burden, and costs of accessing care were not included in the final synthesis because they were considered intended benefits (discussed in the Data Extraction section above)
- Continuity of care refers to uninterrupted care or service across programs, practitioners, and levels over time [121]; as such, consequences under this dimension include findings on care planning and coordination, information sharing between providers, facilitation of multidisciplinary care, frequency of visits, and interpersonal exchange between patients and their providers [121,122]
- Appropriateness is defined as providing care that is person-centered and culturally sensitive, allowing patients to be involved in the decision-making regarding their health choices; when care is appropriate, patients are encouraged to share their experiences and provide feedback on the care they receive without fear of consequences, thus ensuring their dignity and the confidentiality of their health information during and after receiving care [120]
- Safety refers to the reduction or avoidance of harm due to, and during, health care provision or from the environment in which care is delivered [120]; any findings about patients’ safety, staff safety, and medicolegal issues resulting from lack of safety in health care were mapped under this dimension
- Effectiveness refers to achieving the desired clinical outcomes, taking into consideration patients’ perspectives; as such, patient-reported outcomes and patient-reported experiences constitute an essential component of evaluating health care effectiveness [120]; moreover, indicators such as preventable hospitalizations, preventable deaths, and screening and immunization rates are all measures of health care effectiveness [119]; any consequences of telehealth that support or undermine desirable clinical outcomes were mapped under this dimension
- Efficiency and sustainability refers to achieving desirable clinical outcomes at the minimum cost possible and optimal use of health care resources. such as workforce and physical resources, all while innovating to meet increasing health care demands without overstretching the workforce [120]; therefore, findings on telehealth impact on the cost of service, health workforce recruitment and retention, and supporting clinicians were mapped under this dimension

## Results

### Study Selection and Characteristics

**Figure 2:**
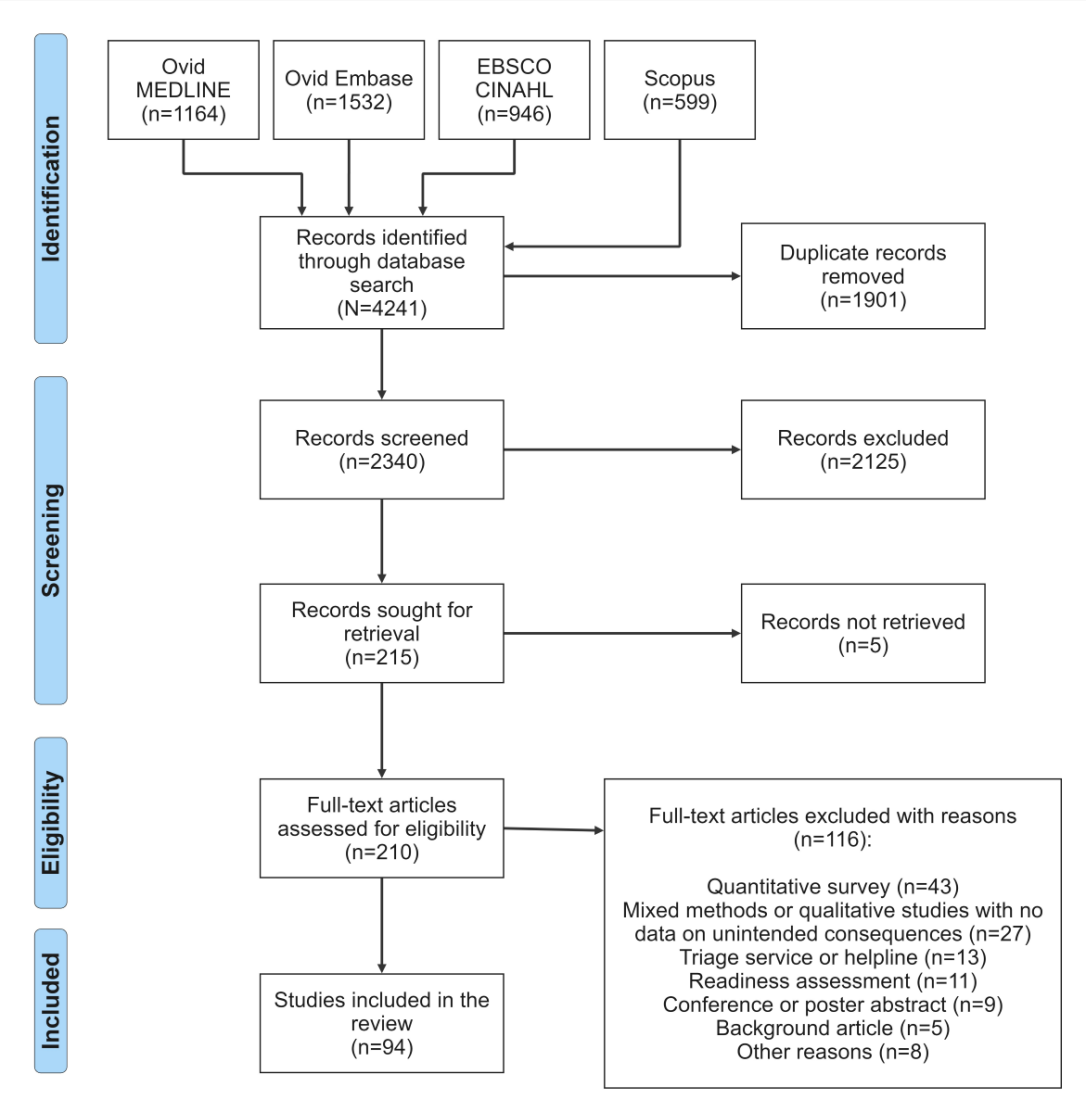
PRISMA (Preferred Reporting Items for Systematic Reviews and Meta-Analyses) search and screening protocol for the literature review on the unintended consequences of telehealth.

Of the 4241 records retrieved, 2340 (55.18%) were screened after 1901 (44.82%) duplicates were removed. After title and abstract screening, 2125 (90.81%) of the 2340 studies were excluded, resulting in 215 (9.19%) studies progressing to full-text review. Studies for which abstracts were unavailable progressed directly to full-text review. In addition, survey studies that fulfilled the inclusion criteria progressed to full-text screening because they could have open-ended sections with relevant data on unintended consequences. At the full-text review stage, of the 215 studies, 121 (56.3%) were excluded, leaving 94 (43.7%) studies (Multimedia Appendix 2) that evaluated telehealth programs in Australia and were eligible for data extraction and analysis. Figure 2 summarized the PRISMA search and screening protocol followed and the number of studies at each stage.

More than half of the included studies (50/94, 53%) were published in the last 3 years (after the onset of the COVID-19 pandemic; Table 1). Nearly a third of the studies (29/94, 31%) were conducted in multiple states or territories across Australia, with the remainder being conducted in Queensland (23/94, 24%), Victoria (18/94, 19%), New South Wales (14/94, 15%), South Australia (5/94, 5%), Western Australia (3/94, 3%), Tasmania (1/94, 1%), and the Northern Territory (1/94, 1%). The fewest studies were conducted in a metropolitan setting (18/94, 19%), with most being conducted in rural or regional settings (32/94, 34%) or in mixed settings (31/94, 33%). Of the 94 studies, 13 (14%) lacked data about whether the study was in metropolitan, rural, or regional settings.

**Table 1:** Number of studies per year (N=94).

Year	Studies, n (%)
2000	2 (2)
2002	1 (1)
2003	1 (1)
2009	1 (1)
2012	5 (5)
2014	4 (4)
2015	5 (5)
2016	5 (5)
2017	1 (1)
2018	3 (3)
2019	5 (5)
2020	10 (11)
2021	18 (19)
2022	32 (34)
2023	1 (1)

Most of the included studies (79/94, 84%) evaluated a synchronous-only model, whereas 16% (15/94) assessed a telehealth model using both synchronous and asynchronous modalities. Almost a third of the included studies (28/94, 30%) examined telehealth in various allied health settings, with the remainder being conducted in other primary care settings (8/94, 9%), mental health (14/94, 15%), or other specialties (oncology 7/94, 7%; palliative care 4/94, 4%; rheumatology 3/94, 3%; emergency medicine 3/94, 3%; surgery 3/94, 3%; hematology 2/94, 2%; geriatrics 2/94, 2%; gynecology and reproductive medicine 2/94, 2%; pulmonology 1/94, 1%; hepatology 1/94, 1%; nephrology 1/94, 1%; endocrinology 1/94, 1%; cardiology 1/94, 1%; anesthesia 1/94, 1%; radiology 1/94, 1%; multiple 11/94, 12%). The majority of the included studies evaluated a patient-to-provider telehealth model (71/94, 76%) whereas the remainder of the studies assessed provider-to-provider models (11/94, 12%), group therapy (3/94, 3%), or a combination of models (9/94, 10%), as shown in Table 2.

**Table 2:** Summary of characteristics of the included studies (N=94).

Study characteristics		Studies, n (%)
**Methods**		
	Mixed methods	50 (53)
	Qualitative	42 (45)
	Quantitative	2 (2)
**States or territories**		
	Queensland	23 (24)
	Victoria	18 (19)
	New South Wales	14 (15)
	South Australia	5 (5)
	Western Australia	3 (3)
	Tasmania	1 (1)
	Northern Territory	1 (1)
	Multiple	29 (31)
**Regions**		
	Regional or rural	32 (34)
	Mixed	31 (33)
	Metropolitan	18 (19)
	Not reported	13 (14)
**Type of telehealth**		
	Synchronous	79 (84)
	Synchronous+asynchronous	15 (16)
**Modalities**		
	Video	54 (57)
	Video+telephone	34 (36)
	Telephone	5 (5)
	Not reported	1 (1)
**Specialties**		
	Allied health	28 (30)
	Mental health (psychology and psychiatry)	14 (15)
	Primary care	8 (9)
	Other specialist services	44 (47)
**Participant groups**		
	Health care providers	46 (49)
	Consumers	23 (24)
	Health care providers+consumers	22 (23)
	Not applicable	3 (3)
**Models of care**		
	Patient to provider	71 (76)
	Provider to provider	11 (12)
	Group therapy	3 (3)
	Multiple	9 (10)

Approximately half of the studies investigated the perspectives of providers in a range of different roles (46/94, 49%), nearly a quarter examined the views of consumers and carers (23/94, 24%), and 23% (22/94) included the perspectives of both consumers and health care providers. A few of the studies (3/94, 3%) did not recruit participants: they either examined existing documents or analyzed routinely collected data. Only 14 (15%) of the 94 studies used theoretical frameworks or models to underpin the study design and analysis, with the Nonadoption, Abandonment, Scale-up, Spread, and Sustainability Framework [123] being the most commonly used framework among these 14 studies (n=3, 21%).

### Unintended Consequences of Telehealth in Australia

**Figure 3:**
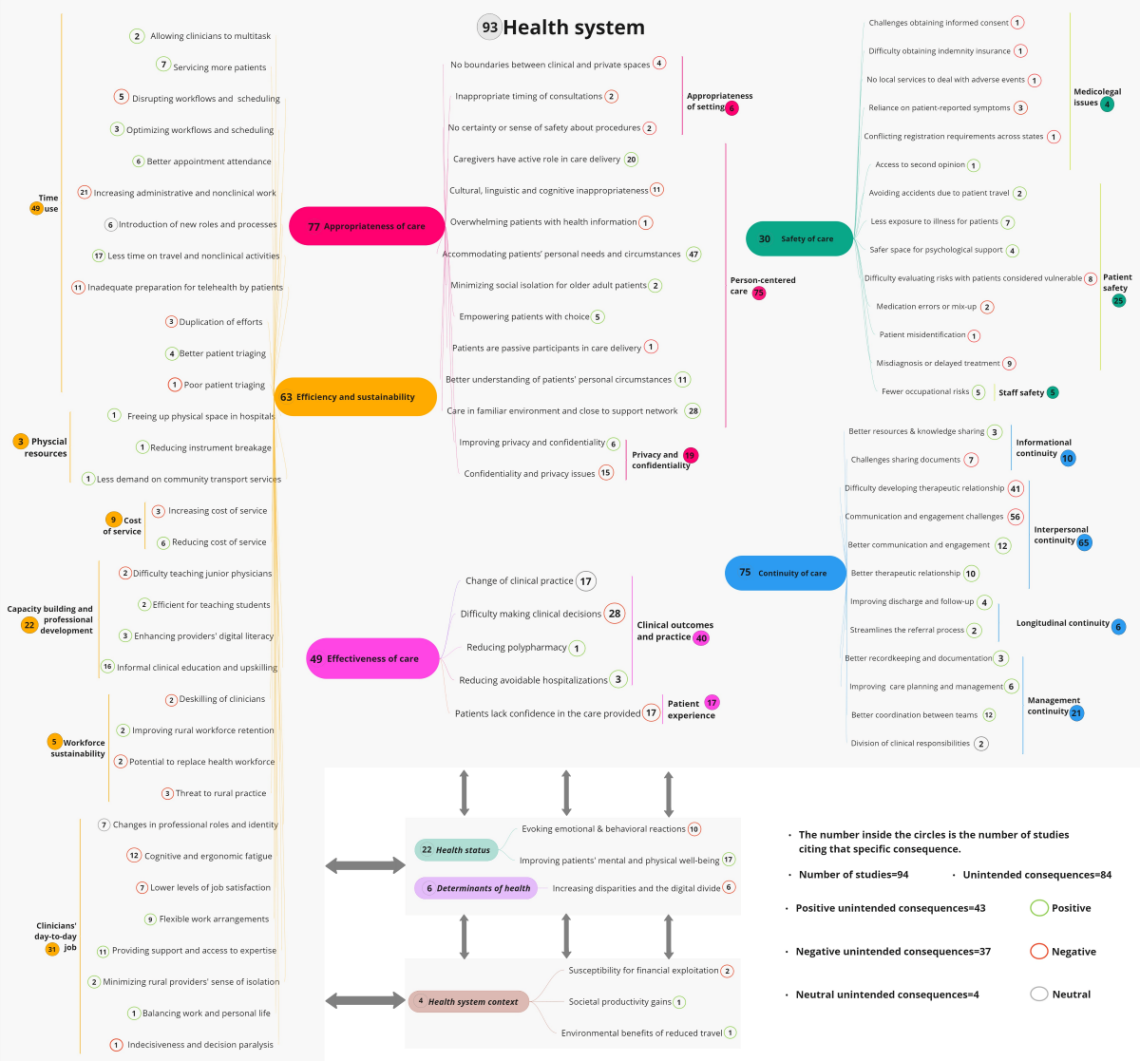
The unintended consequences of telehealth across the Australian Health Performance Framework (AHPF) domains and dimensions and the newly devised subcategories across each dimension. The AHPF domains are interrelated; for example, health status and determinants of health are influenced by, and influence, the dimensions of the health system and the health system context, while the health system influences the broader context within which it exists and vice versa.

The unintended consequences of telehealth extracted from the included studies were mapped into the 4 domains of the AHPF, as described in the following subsections and summarized in Figure 3.

#### Health Status

Under this domain, nearly a fourth of the studies (23/94, 24%) reported unintended consequences of telehealth associated with health status, with most of the studies (17/23, 74%) reporting positive consequences; for example, 71% (12/17) of these studies reported that telehealth improved patients’ mental and physical well-being, mostly because patients were in the comfort of their homes where they felt more relaxed, less vulnerable, and in control compared to being in an unfamiliar or clinical environment [8,22-32]. Some of the studies (6/17, 35%) highlighted other contributing factors to this improved well-being, such as the reduction in travel-induced anxiety and fatigue [24,33-36], as well as the social support resulting from connecting with people with similar experiences or challenges in group therapy [37]. However, several of the studies (10/23, 43%) highlighted some negative unintended consequences of telehealth, such as evoking behavioral reactions in children [25,28,38] and negative emotions from seeing one’s image on the screen [31,32,34,39], including for those with, for example, eating disorders [31,32] or hearing aids [39]. In some of the studies (5/23, 22%), telehealth was found to provoke anxiety and discomfort in patients [6,25,40,41].

#### Determinants of Health

Of the 94 studies, 6 (6%) reported a potential negative impact of telehealth on individuals’ socioeconomic status and other determinants of health. Specifically, 5 (83%) of these 6 studies reported concerns about the potential of telehealth to increase the digital divide due to the increased financial burden on consumers, resulting from internet and equipment costs [40,42-45]. In 2 (33%) of the 6 studies, lack of digital literacy [44], particularly among the older populations [30], was highlighted as another factor that could increase this digital gap.

#### The Health System Context

The findings of 4 (4%) of the 94 studies under the health system context emphasized telehealth’s impact on factors external to, and generally beyond, the direct influence of the health system. Although 2 (50%) of these 4 studies reported positive outcomes of telehealth associated with this domain, such as societal productivity gain [46] and the environmental benefits of reduced travel [47], 2 (50%) studies highlighted the potential for financial exploitation through telehealth [48,49].

#### The Health System

This domain accounts for a majority of the reported unintended consequences of telehealth synthesized in this review. Of the 94 included studies, 93 (99%) reported findings with unintended consequences falling across the dimensions of the health system domain as detailed in the following subsections.

##### Accessibility

There were no reported unintended consequences under this dimension because all extracted outcomes were related to reducing the cost and time of accessing care and, as such, were considered intended benefits, as indicated earlier.

##### Safety

In 30 (32%) of the 93 studies, the safety of care provision was shown to be impacted by telehealth, with more than half (n=18, 60%) noting 9 negative consequences. In 10 (56%) of these 18 studies, telehealth was reported to jeopardize patient safety due to patient misidentification [45], medication errors [41,45], the risk of misdiagnosis [29,45,47,50-55], or delayed treatment [29,45]. Of these 10 studies, 6 (60%) highlighted some factors that give rise to these risks of misdiagnosis and delayed treatments in telehealth, such as lack of nonvisual cues [45,47,51,52], patients not preparing properly for a telehealth consultation as they would for an in-person consultation [45], and the lack of a physical examination [45,51,53,54]. According to 8 (44%) of the 18 studies noting negative consequences, the lack of a physical examination was believed to have led to overreliance on patient-reported symptoms [45,47,51,53,55], potentially increasing medicolegal liabilities [50,56,57]. Moreover, the findings of 3 (17%) of the 18 studies suggest that the use of telehealth could lead to medicolegal liabilities due to conflicting state registration requirements, potentially causing uncertainty surrounding professional liability in case of clinical incidents [58]; a lack of local services to deal with adverse events [50]; and challenges obtaining informed consent from patients due to miscommunication challenges [57]. Difficulties in evaluating, identifying, and addressing risk through telehealth were reported in 8 (44%) of the 18 studies involving patients considered vulnerable, including those who have experienced domestic violence or are at risk for suicide [31,32,40,45,52,59-61].

By contrast, 16 (53%) of the 30 studies noted 5 positive telehealth outcomes associated with health care safety. Of these 16 studies, 5 (31%) demonstrated that telehealth improved staff safety by reducing occupational risks associated with home visits [24,50,56], work-related travel [50], exposure to illness [62], and carrying work equipment [22]; and 13 (81%) highlighted the potential of telehealth to improve patient safety by reducing accidents resulting from patient transportation [50,63] and minimizing exposure to illness [29,36,64], which was particularly beneficial for patients with a compromised immune system [7,22,65,66]. According to 4 (25%) of the 16 studies, telehealth provided a safe space for psychological support for patients considered vulnerable [30,31,40,67]. Finally, the findings from 1 (6%) of the 16 studies suggested that telehealth has the potential to reduce medicolegal liabilities by reducing accidents while transporting patients, minimizing the risk of misdiagnosis by providing additional expertise through second opinions, and providing better incident auditing [50].

##### Appropriateness

Of the 93 studies, 77 (83%) documented several unintended consequences under the appropriateness dimension in terms of person-centered care, the appropriateness of the setting, and the privacy and confidentiality of patients.

Of these 77 studies, 75 (97%) reported telehealth consequences—primarily positive—related to person-centered care. Several telehealth benefits were documented in numerous studies (60/75, 80%), such as minimizing social isolation for older adult patients [66,68], allowing patients to receive care in a familiar and comfortable environment [7,8,23-26,28-30,32,33,64,68-77], allowing patients to receive care close to their support network [27,69,78,79], and accommodating patients’ personal needs and circumstances [6-8,22,24,26,28-30,32,34-37,40,43,45,47,48,50-52,55,59-61,64,65,67-70,75-77,80-91]. These factors helped patients to feel relaxed and encouraged them to open up more easily [36,52,70-72,80]. In 5 (7%) of the 75 studies, telehealth was reported to empower patients and reinforce their autonomy [31,39,50,77,82], in contrast to findings from a study in which several patients described their role as “passive” during telehealth consultations compared to in-person consultations [51].

In several of the studies (11/75, 15%), clinicians shared their views on how telehealth allows them to see patients in their personal spaces, enabling them to provide more personalized care [8,32,42,55,61,67,73,75,80,82,92]. A number of studies (20/75, 27%) highlighted that telehealth facilitated the involvement of family members and carers and supported them in having an active role in care delivery [8,22,25,26,29,32,34,38,56,62,63,67,77,79,85,93-97]. However, this increased involvement was viewed differently by clinicians and carers; some carers complained about the increased burden and responsibilities [22,38,56,93]. By contrast, clinicians were anxious about losing control due to reliance on carers to perform tasks they would have otherwise performed themselves in an in-person consultations if not for the limitations of delivering care via telehealth [25,94]. In addition, clinicians complained about parents’ noncompliance to given instructions, which affected the quality of care provided during telehealth consultations [25,94].

A number of studies (12/75, 16%) pointed out other consequences of telehealth that undermined person-centered care. In 7 (58%) of these 12 studies, clinicians raised concerns about the cultural, cognitive, and social inappropriateness of telehealth for various groups such as Indigenous communities [47,83], patients with mental or cognitive impairment [49,83,97], patients with hearing impairments [29,30], patients who were acutely unwell [83,97], and rural and remote communities where an essential part of delivering care to patients is knowing their social circumstances [44]. Furthermore, according to the findings from 8 (67%) of the 12 studies, communication challenges were reported in telehealth consultations involving non–English-speaking patients or patients who required interpreters to be present [29,30,49,52,57,64,83,98]. In 1 (8%) of the 12 studies, clinicians shared their concerns that telehealth patients were being overserviced and overloaded with information relating to their health [42].

Of the 77 studies, 4 (5%) reported findings about the appropriateness of telehealth as a health care delivery setting compared to physical or brick-and-mortar settings. In 2 (50%) of these 4 studies, patients shared that telehealth removed the sense of safety and certainty that came from being surrounded by health professionals in physical settings where they felt more supported and had multiple opportunities to raise any concerns or issues [31,51]. A different study reported scheduling confusion and inappropriate timing of telehealth consultations [29], which was supported by findings from another study in which patients shared their frustration that clinicians prioritized in-person consultations and fitted telehealth consultations in between, leading to scheduling confusion and inappropriate timing of consultations [64].

Telehealth consequences related to patient privacy and confidentiality were documented in 19 (25%) of the 77 studies, with negative consequences being reported in most of them (n=16, 84%). Of the 19 studies, 8 (42%) reported clinicians’ concerns about patients’ privacy and confidentiality in telehealth consultations due to lack of private spaces to discuss sensitive matters, especially for patients considered vulnerable [28,40,42,45,50,52,54,89]. Similarly, in 5 (26%) of the 19 studies, telehealth was viewed as blurring the boundaries between private and clinical spaces [28,29,31,32], thereby invading the privacy of both patients [28,32,89] and clinicians [29]. In 5 (26%) of the 19 studies, patients were reportedly concerned about their privacy and confidentiality in telehealth consultations due to a distrust of technology [32], fear of cybersecurity breaches [64], and the possibility that other people might overhear their conversations [29,31,36]. According to 2 (11%) of the 19 studies, errors such as clinicians calling the wrong person [45] or patients mistakenly joining other patients’ consultations [33] were reported as jeopardizing privacy during telehealth consultations. In addition, 2 (11%) of the 19 studies documented clinicians’ views on the inappropriateness of a telehealth physical examination because patients get undressed in front of the camera [47,54].

These issues notwithstanding, 6 (32%) of the 19 studies reported positive consequences of telehealth associated with patient privacy and confidentiality. Of these 6 studies, 4 (67%) reported that telehealth allowed patients to seek care anonymously—in contrast to in-person care where patients may be seen or questioned by acquaintances when seeking care [52,61,83,99]. This feature of telehealth was particularly valued in small and Indigenous communities and when the topic was sensitive because patients had the option to seek care outside their community [61]. In 2 (33%) of the 6 studies, patients shared that telehealth allowed them to talk freely in the privacy of their homes compared to hospitals and other clinical settings where there is a risk of other people overhearing their conversations [72], all while controlling the level of exchange they prefer with the ability to turn the video on and off [31].

##### Effectiveness

Of the 93 studies, 49 (53%) reported telehealth’s unintended consequences pertaining to the effectiveness of care in terms of clinical practice and clinical outcomes, as well as patient experience. Documented in 28 (57%) of the 49 studies, the most cited negative unintended consequence of telehealth under this dimension was the clinicians’ inability to make clinical decisions when delivering care via telehealth, potentially impacting clinical outcomes [22,24,25,28-30,32,44,45,47,51-56,59, 62,65-67,80,84,92,97,98,100,101]. The factors contributing to these challenges in clinical decision-making were presented in a number of studies (26/49, 53%), such as a lack of nonverbal cues as well as communication challenges [22,24,25,28-30,45,51,52,56,66,80,92], the inability to examine patients in person [29,30,32,45,47,53-56, 59,65,66,84,92,97,98,101], and a lack of local and contextual understanding of the patients’ circumstances, particularly when providing care via telehealth to rural and regional patients [44,100].

According to a number of studies (17/49, 35%), telehealth forced clinicians to change and adapt their clinical practices and processes to fit the new mode of delivery [8,22,29,31,45,49,50,52,56,59-61,66,91-93,95]. In several cases (10/17, 59%), this adaptation was believed to be a consequence of the inherent limitations of telehealth, such as the inability to examine patients in person [29,92], a lack of nonverbal cues that affect communication and rapport building [8,45,91,92,95], and the inability of the health professional to guide and provide physical support to patients or encourage participation [22,60,93]. In addition, in 2 (12%) of the 17 studies, clinicians adapted their clinical practices to ensure patients’ privacy during telehealth consultations [91] and to mitigate the risk of medicolegal liabilities [50]. Regardless, in a few studies (4/49, 8%), telehealth was viewed as potentially improving clinical effectiveness by reducing avoidable hospitalizations [63,83,102] and polypharmacy [63].

Unintended consequences in relation to patient-reported experiences of receiving care via telehealth were documented in 17 (35%) of the 49 studies in which patients shared concerns and a lack of confidence in the care they received and questioned whether it was as thorough and accurate as in-person care [6,7,25,29,32,33,35,36,38,41,44,45,51,64,69,84,101]. Their primary concerns included that clinicians may have missed something when delivering care via telehealth due to the lack of an in-person physical examination and physical interactions [6,7,33,35,36,38,44,51,64,69,84,101], communication challenges [6,32,44,64], and telehealth consultations being rushed by clinicians [6,36,41,64].

##### Continuity of Care

Of the 93 studies, 75 (81%) documented telehealth’s unintended consequences associated with the 4 categories of continuity of care: informational continuity, interpersonal continuity, longitudinal continuity, and management continuity.

On the basis of the findings of 7 (9%) of these 75 studies, informational continuity was hindered by the use of telehealth, such as when sharing documents, test results, and prescriptions with patients [29,41,69,98,103] or with other clinicians to assist in clinical decision-making [29,65,104]. However, these findings are in contrast to what was reported in a study in which clinicians reported better access to, and sharing of, resources [22]. Likewise, in 2 (3%) of the 75 studies, telehealth was believed to improve informal knowledge sharing between providers to support care delivery [50,105].

In a number of studies (18/75, 24%), telehealth was viewed as facilitating case management and multidisciplinary care because it improved care coordination and communication between teams [30,32,42,48,50,63,79,84,86,88,102,105]; supported better recordkeeping and documentation [30,50,103]; and improved care planning [24,32,45,55,64,65], which was particularly beneficial in the management of chronic and complex conditions [45,64,65]. In addition, the findings from 6 (8%) of the 75 studies suggested that telehealth supported longitudinal continuity across the continuum of care by streamlining the referral process [63,100], allowing clinicians to make informed discharge decisions [24,84], and improving the follow-up experience for patients [45,47]. Finally, a division of clinical responsibilities, role reassignment, and changes in power dynamics were noted as unintended consequences of telehealth when multiple clinicians were involved in case management [50,106].

Of the 75 studies, 65 (87%) reported unintended consequences of telehealth related to interpersonal continuity of care in terms of interpersonal exchange and therapeutic relationship building. Specifically, in 56 (86%) of the 65 studies, telehealth was viewed as hindering effective communication and patient engagement [7,22-25,28-33,35-38,40-45,47-49,51,52,55-58, 60-62,64,66,67,71,75,76,81,83,84,90,91,93-99,101-103,107,108], largely due to the lack of nonverbal information [7,22,24,25,28-33,35-38,40-45,47-49,51,52,55-58,61,64,66,71,76,81,84,90,91,93,95,101,107,108] and, to a lesser extent, due to the increase in distractions during telehealth consultations compared to in-person consultations [22,25,32,38,40,62,67,75,91,93,94,96], particularly in the case of children or young patients [22,25,32,38,40,62,67,75,93,94,96]. Nevertheless, these findings are in contrast to those reported in 12 (18%) of the 65 studies that documented an improvement in communication and patients’ engagement in telehealth [22,28,34-36,40,50,52,70,71,84], primarily due to patients being comfortable in their homes and more inclined to open up about sensitive health issues [36,40,52,70,71], fewer distractions for both patients and clinicians [22,34,75], and the increased interest in technology among children [35]. Of the 12 studies, 2 (17%) suggested that patients opened up more easily and were less inhibited over telehealth because they were unaffected by clinicians’ presence and any nonverbal cues that may have signaled patients to stop talking [50,84].

In 41 (63%) of the 65 studies reporting unintended consequences of telehealth related to interpersonal continuity of care, telehealth interactions were noted as impersonal, thus undermining rapport building and affecting the therapeutic patient-provider relationship [6,7,22,24,28-31,35,36,38-42,44,45,47-52,56,60,61, 64,68,76,80,81,84,90,91,93-95,97,98,101,107]. However, contrary to these findings, 10 (15%) of the 65 studies reported better therapeutic relationship building in telehealth as a consequence of the improved communication and engagement in telehealth [42,75] as well as the increased frequency of contact between patients and their providers over extended periods of time [26,29,68,71,81,84,85,99].

##### Efficiency and Sustainability

Of the 93 studies, 63 (68%) reported unintended consequences of telehealth associated with the efficiency and sustainability of health care in terms of its impact on health workforce and resources use. According to the findings from 11 (17%) of these 63 studies, health professionals reported that telehealth supported them in their daily tasks by providing access to a second opinion or specialized expertise [40,44,50,58,68,77,83,85,108-110] and thereby minimized their feeling of isolation [50,109], potentially improving rural workforce retention [44,50]. In addition, other unintended benefits of telehealth for clinicians were documented in many of the studies (11/63, 17%), such as improved digital literacy and confidence using technology [37,68,111], improved work-life balance [22], and increased flexibility in terms of the time and location of consultations [24,29,37,40,68,82,83,88,94]. However, according to a study, this increased flexibility made possible by telehealth caused decision paralysis for clinicians [31].

On the basis of the findings from 11 (17%) of the 63 studies, telehealth caused health care providers cognitive fatigue [22,29,31,32,45,51,52,55,80,93,94] due to the increased energy demand to maintain patient engagement [22,32], the higher concentration required due to the lack of nonverbal cues [29,52], the new routines and administrative work resulting from delivering care via telehealth [93,94], and the constant anticipation of what comes next and the adaptation to accommodate these new routines and changes [31,45,51]. In 1 (2%) of the 63 studies, telehealth was reported to have caused ergonomic fatigue due to the extended periods providers spent sitting when providing care via telehealth [92]. As documented in 7 (11%) of the 63 studies, telehealth was viewed as less rewarding and less satisfying to clinicians than in-person care delivery due to the absence of the personal touch and therapeutic connection [29,51,53,55,80,84,92]. Finally, 7 (11%) of the 63 studies noted a consequence of telehealth where it was reported to affect clinicians’ roles and professional identity [49,50,85,92,93,106,110].

A number of studies (23/63, 37%) documented both positive and negative unintended consequences of telehealth associated with workforce sustainability and professional development. In particular, 2 (9%) of the 23 studies reported concerns about telehealth deskilling metropolitan clinicians providing care to rural regions via telehealth due to a lack of the hands-on experience necessary to build clinical skills [78] as well as rural clinicians due to a lack of opportunities to practice [57]. Thus, as noted in 3 (13%) of the 23 studies, telehealth could potentially threaten the viability and sustainability of rural practice by leading to the loss of rural patients to metropolitan providers [44,50,57]. Furthermore, in 2 (9%) of the 23 studies, telehealth was viewed as a technological replacement for health professionals [81], potentially leading to shortages in the workforce and exacerbating health care accessibility issues in the long term [50]. Nevertheless, as evidenced by a number of studies (18/23, 78%), telehealth was reportedly efficient for student supervision [68,82], improved clinicians’ access to informal training and education, and supported capacity building and continuing professional development for rural health professionals [32,42,50,58,63,77-79,83,85,88,99,100, 102,105,112]. However, 2 (9%) of the 23 studies presented contradictory findings because several clinicians shared difficulties teaching junior physicians and students via telehealth [29,80].

Of the 63 studies, 51 (81%) documented 12 unintended consequences pertaining to the impact of telehealth on the use of resources in health care. On the basis of evidence from a number of studies (29/51, 57%), telehealth enhanced productivity, improved the timeliness of care, and enabled better use of physical resources; for instance, in 20 (69%) of the 29 studies, telehealth was reported to have saved clinicians’ time [29,49,56,58,88,102], particularly time spent on commuting or traveling to clinical facilities or patients’ homes [7,22,50,62,65,68,82,83,89,103,106] and social and nonclinical activities [89], allowing them to service more patients [24,37,62,66,68,82,88]. Other reported telehealth benefits associated with time use included allowing providers to multitask [43,92], improving appointment scheduling [22,82] and attendance by patients [22,24,29,68,84,98], speeding up the referral process [100], and streamlining patient triage [24,55,65,107]. According to 6 (12%) of the 51 studies, telehealth can potentially reduce health care system expenditures due to reduced cancellation rates [22], saving clinicians’ travel time [58,79,83], avoiding unnecessary patient transportation [112], and reducing reimbursements of patient travel [88]. Finally, in 3 (6%) of the 51 studies, telehealth was reported to have resulted in efficient use of the physical resources essential for health care delivery because it freed up space in hospitals [92], lowered demand for community transport services [68], and reduced instrument breakage [22]. By contrast, many studies (34/51, 67%) noted unintended drawbacks of telehealth related to the degradation of efficiency in health care delivery. As evidenced in 21 (62%) of the 34 studies, this degradation in efficiency was primarily due to the increased time spent by clinicians on administrative and nonclinical workloads [22,31,37,43,45,47,49,51,53,55,59,62,65,68,81,83,93,94,96,104,112]. Furthermore, 16 (47%) of the 34 studies reported other sources of inefficiencies in telehealth, such as the duplication of efforts by both patients and clinicians [41,45,53]; poor triaging [80]; impaired and disrupted workflows [29,45,47-49,64]; and patients not preparing properly for a telehealth consultation as they would for an in-person consultation, wasting the clinician’s time and theirs [29,30,32,45,49,51,52,67,76,80,84]. According to 3 (9%) of the 34 studies, inefficiencies in telehealth care delivery added to health care delivery costs, making telehealth cost-ineffective and financially unviable for providers [53,55,57]. Finally, the findings from 6 (18%) of the 34 studies noted telehealth’s impact on health care efficiency because it added new processes and workflows or adapted existing ones [30,45,49,93,96,111].

## Discussion

### Principal Findings

Our synthesis of studies evaluating telehealth in Australia suggests that there are both positive and negative unintended consequences, often affecting the same aspect of health care. This conclusion was particularly evident in how telehealth impacted patients’ privacy because several of the included studies (4/94, 4%) documented a positive impact of telehealth on privacy in small and Indigenous remote communities because it allowed anonymity [52,61,83,99]. By contrast, other included studies (8/94, 9%) reported privacy and confidentiality concerns, particularly for groups considered vulnerable, such as children and those who have experienced domestic violence [28,40,42,45,50,52,54,89], as a result of the blurred boundaries between personal and therapeutic or clinical spaces. These findings are in line with another issue highlighted in our review that telehealth compromised the safety of domestic violence victims and patients who are at risk of suicide because it was challenging to assess risks in the surrounding environment via telehealth [32,40,45,52,59-61]. These issues notwithstanding, a number of studies (13/94, 14%) in our review reported positive consequences of telehealth on patient safety due to various factors appertaining to the provision of care at home and in a familiar environment, as well as the elimination of unnecessary travel [7,22,29-31,36,40,50,63-67].

While the findings from our review present telehealth as a model that largely supports a person-centered approach to care delivery, the included studies also recorded challenges undermining person-centered care delivery via telehealth. Notably, telehealth was often reported to be inappropriate and unfit for the personal and cultural needs of certain groups, such as culturally and linguistically diverse communities [29,30,49,52,57,64,98], Indigenous patients [47,83], and patients with cognitive and hearing impairments [29,30,49]. The positive impact of telehealth on longitudinal and management continuity of care featured prominently throughout our review. However, telehealth was viewed as a hindrance to interpersonal continuity of care and challenging for rapport building, and as such, telehealth seems better suited as a complementary model rather than a substitute for in-person care because strong interpersonal therapeutic relationships are necessary for safe, efficient, and person-centered care.

At times, telehealth resulted in neutral consequences with no clear intrinsic positive or negative attributes. Identifying these consequences can provide significant leverage to influence and improve care delivery via telehealth because they were often intermediate consequences that could lead to long-term positive or negative outcomes; for example, we categorized the introduction of new workflows and processes as a neutral consequence because it is not inherently positive or negative. However, the findings from multiple studies (5/94, 5%) in our review indicated increased workload and inefficiencies in service delivery due to these new workflows and processes [45,49,59,65,93]. Alternatively, according to 1 (1%) of the 94 included studies, telehealth provided an opportunity to redesign service delivery because it improved existing processes [105]. Another noteworthy issue is the differentiation between actual and potential outcomes because some of the studies (12/94, 13%) highlighted several potential unintended consequences—in addition to actual consequences—which were often voiced as concerns by stakeholders. These were often long-term consequences associated with workforce sustainability [50,57,78,81], the determinants of health [30,40,42-45], and the broader financial context within which the health system operates [48,49]. Including these potential outcomes in our review enables policy makers and health care planners to anticipate and prepare for future challenges and opportunities, facilitating proactive risk mitigation and benefit maximization, which is particularly important in the rapidly evolving field of telehealth.

### International Relevance

While our study primarily focused on the Australian context, the unintended consequences of telehealth implementation identified in this review have broader international relevance. Many of our findings resonate with research conducted in countries with similar health care systems or those facing comparable geographic challenges. These findings suggest that certain unintended consequences of telehealth may be inherent to the modality itself rather than being solely context specific; for instance, a recent review, which included studies from multiple countries, corroborates our findings on telehealth’s ability to empower patients, foster a perceived safe environment, and enhance family involvement [124]. While the review focused on telehealth for pain management, the findings from our review show that these patterns extend beyond pain management to encompass other specialties such as geriatric care, mental health services, and allied health disciplines, underscoring telehealth’s capacity to facilitate a person-centered approach to health care delivery. Furthermore, the potential of telehealth to address significant contributors to clinicians’ burnout, as highlighted in a Canadian study [125], corroborates our findings on the unexpected positive impacts of telehealth on clinicians’ work-life balance, underscoring the importance of considering telehealth not only as a tool for patient care but also as a potential strategy for improving health care workforce sustainability.

Conversely, our review also uncovered negative unintended consequences of telehealth that align with international findings. The cognitive and physical fatigue experienced by clinicians due to increased administrative workload and heightened concentration demands, as identified in our Australian-focused review, are mirrored in an international scoping review [126]. These findings suggest that the challenges of adapting to telehealth modalities are a universal experience for health care providers across different health care systems. More critically, our findings highlight telehealth’s potential to exacerbate existing health care disparities, a concern corroborated by research from the United States, suggesting that specific populations—particularly older adults, those with lower socioeconomic status, and non–English-speaking patients—faced challenges in accessing telehealth services during the COVID-19 pandemic [127]. This alarming trend underscores the crucial need to prioritize equity of access in telehealth implementation, regardless of the health care system context.

While many of our findings have international parallels, some of our results may be more specific to the Australian context; for instance, our observations regarding providing support and informal training for rural clinicians through telehealth might be particularly relevant to Australia’s unique geographic and demographic circumstances. While countries such as Canada or the United States might face similar rural health care challenges, the specific implementation and impact of telehealth in these areas may differ due to variations in health care systems, policies, and cultural factors. Therefore, caution is needed when interpreting and translating these findings to other health care systems.

### Implications for Practice and Policy Making

One challenge in telehealth consultations that was featured repeatedly in multiple studies (17/94, 18%) in our review is the inability to examine patients in person [29,30,32,45,47,53-56,59,65,66,84,92,97,98,101]. Some of the reported ways to mitigate the risk of wrong assessment due to this lack of in-person physical examination included selecting appropriate patients and cases for telehealth [50], spending extra time eliciting information from patients [45], lowering the diagnostic threshold [45,50], and conducting initial in-person assessments to gain a better understanding of the patient’s condition and circumstances [50,54]. Evidently, clinicians rely on their discretion when determining which cases or conditions are appropriate and safe for telehealth and what risk mitigation strategies to use. Professional bodies should frequently release evidence-based guidelines on which aspects of health care delivery and what cases are appropriate and safe for telehealth to assist clinicians in decision-making and ensure standardization. Recently, the Medical Board of Australia released telehealth guidelines incorporating feedback from professionals and the community [128]. However, these guidelines lack details on cases, conditions, or aspects of care that are more suited to telehealth than in-person care; in addition, they do not provide recommendations on risk mitigation strategies beyond the technological and procedural aspects and routine precautions that apply to the provision of care in general, regardless of modality.

Adopting a hybrid mode where providers combine telehealth and in-person care may lead to higher job satisfaction for clinicians because this mode can offer the rewarding feeling of providing care face-to-face that is lacking when delivering care via telehealth [29,51,53,55,84,92,93], while balancing work and personal life [22]. As such, the hybrid delivery mode could reduce burnout and help attract and retain health professionals in rural and regional areas that experience health workforce shortages. However, an unintended consequence of telehealth that was identified in our review is its potential to threaten rural practice [50,57], necessitating safeguards to ensure that rural and regional areas receive funding, training opportunities, and the on-the-ground workforce necessary to ensure the quality and safety of health care delivered to these communities. Furthermore, the fact that challenges were reported when delivering care to rural and regional patients due to a lack of context and local knowledge [44,100] underscores the need for clinicians to dedicate time to acquiring contextual knowledge and understanding patients’ specific circumstances to build solid therapeutic relationships and ensure the relevance of the clinical advice provided.

One issue that was featured in our review is the ambiguity regarding clinical roles and responsibilities when multiple clinicians are involved in care delivery via telehealth [50,58,106], suggesting the need for policy or laws to govern care provision via telehealth when multiple clinicians are involved, especially across various states or territories. We will need to specify roles and clinical responsibilities and be ready to deal with any medicolegal issues that may arise in such situations. Finally, perhaps the most concerning finding is the potential of telehealth to widen the digital divide, increasing disparities in health care access among specific groups such as older patients or those with limited digital literacy skills [30,45]. Initiatives to improve patients’ digital literacy [129,130] and provide patient information and guidelines on preparing for telehealth consultations [131,132] may support inclusive and equitable health care access via telehealth.

### Strengths and Limitations

The strength of this review is the inclusion of diverse qualitative, quantitative, and mixed methods studies from 4 databases, ensuring a comprehensive account of the unintended consequences of telehealth for the Australian health system. Our review translated these findings on the unintended consequences of telehealth into the AHPF dimensions, outlining a framework for assessing telehealth impact across the various health system domains and beyond and enabling comparison and standardization. Moreover, by systematically reviewing and categorizing unintended consequences, our study provides insights into which consequences are most frequently reported in the literature. This quantification helps prioritize areas for further research and policy attention, especially because recent policy changes and government funding have signaled a push toward incorporating telehealth as a standard modality of health service provision [133]. Thus, evidence-based insights such as those generated by our review are crucial for offering detailed recommendations for practitioners and policy makers on improving health care delivery via telehealth. To our knowledge, this review is the first attempt to synthesize evidence on the unintended consequences of telehealth, not only in Australia but also internationally.

While a CIS has strong characteristics such as flexibility and interpretive freedom in the inclusion and synthesis of results, it can be a double-edged sword in cases such as our review where there are few to no published studies explicitly focused on the topic. Therefore, the evidence extracted from the included studies depends entirely on the authors’ interpretation of what can be considered an unintended consequence of telehealth, possibly leading to the exclusion of some relevant evidence. In our review, this negative impact was mitigated by the significant number of studies included, ensuring comprehensiveness. Furthermore, to minimize the impact of subjectivity when extracting data, we used explicit definitions for what constituted an *unintended* versus an *intended* consequence to guide our data extraction.

While our review identified overarching patterns and themes that cut across different telehealth modalities and specialties, a limitation of this study is the broad categorization of telehealth modalities without distinguishing between videoconferencing and telephone-based telehealth or between specialty-related consequences and the generalization of videoconferencing technologies. This approach may obscure important nuances specific to different modalities, technologies, or specialties; for instance, videoconferencing may present unique challenges related to technology use and nonverbal communication that are not present in telephone-based telehealth. These unique challenges underscore the need for more detailed analysis. Similarly, certain specialties may encounter distinct unintended consequences based on their specific care requirements.

Moreover, we did not distinguish between the various models of videoconferencing telehealth, such as dedicated videoconferencing units with pan, tilt, and zoom capabilities; computer-based videoconferencing; and mobile phone–based videoconferencing apps. These technologies may have unique characteristics that could lead to specific unintended consequences. While our approach offers valuable insights into general trends across telehealth implementations, it may not capture these modality-, specialty-, or technology-specific nuances.

### Future Directions

There is still a dearth of studies focusing solely on the unintended consequences of telehealth. One plausible explanation could be that telehealth uptake only expanded recently, and long-term outcomes often take time to emerge. In addition, designing a study to investigate unintended consequences using conventional methods can be challenging. To capture the broader impact of telehealth and grasp the complex dynamics that could lead to the emergence of these consequences, studies with designs suited to study the complex nature of telehealth implementation are needed. Recent work proposed a complexity-informed and system-thinking–guided approach to investigate such unintended consequences, emphasizing the interconnectedness between the various actors involved in telehealth implementation [13]. Such theoretical frameworks can provide a holistic understanding of the situation compared to the linear reductionist approach that is often used to study and evaluate telehealth programs.

There is little to no research examining the impact of telehealth beyond the health system and how telehealth influences, and is influenced by, factors such as the determinants of health and the broader health context, such as financial and societal aspects. We found that only 6 (6%) of the 94 studies touched upon the issue, and it was a secondary observation briefly noted by participants [30,40,42-45]. One noteworthy finding of our review is the potential of telehealth to threaten rural practice [50,57], suggesting a need for further research that investigates the impact of telehealth on rural workforce sustainability and rural practice viability. This is particularly important given the complexities of rural health and rural practice [1,134] and the growing perception of telehealth as a favorable solution to health care inaccessibility in rural areas [50,135-137]. Furthermore, additional research is necessary to investigate the safety of health care provision via telehealth and to examine and compare the effectiveness of telehealth in terms of clinical outcomes across various settings, population groups, and specialties.

Further research is needed to address some of the aforementioned limitations regarding the broad categorization of telehealth modalities. Such research should focus on conducting comparative analyses between different telehealth modalities and exploring how unintended consequences manifest across various specialties. These focused studies could provide a more granular understanding of how the choice of the telehealth modality and the specific medical context influence the nature and prevalence of unintended consequences. This would further enhance our understanding of telehealth implementation and guide more tailored strategies for mitigating potential negative outcomes in specific telehealth contexts. Future research could also build on our findings by conducting a more granular analysis of unintended consequences associated with specific videoconferencing technologies. Such studies could explore how factors such as image quality, ease of use, mobility, and specific features (eg, screen-sharing and recording capabilities) influence the nature and prevalence of unintended consequences. Moreover, as telehealth technologies continue to evolve rapidly, ongoing research will be crucial to understanding the implications of emerging videoconferencing tools and platforms and exploring consequences related to augmented reality features, artificial intelligence–assisted communication, or integration with other medical devices and systems.

### Conclusions

The unintended consequences of telehealth synthesized in our review provide a framework for understanding the full impact of telehealth across the health care system and beyond. Identifying these consequences offers various opportunities to more fully leverage the advantages of telehealth while mitigating any potential harm, ultimately sustaining its adoption beyond the COVID-19 pandemic for safe and high-quality care across different settings and population groups. Planning and implementing a telehealth project is a complex undertaking, and while it is not entirely possible to plan and anticipate every possible unintended outcome, the consequences presented in our review provide a road map for planning, implementing, and scaling up telehealth projects to realize their full potential.

## Appendix

Multimedia Appendix 1 Search strategy.

Multimedia Appendix 2 List of selected characteristics of the included studies and extracted unintended consequences.

## Abbreviations

AHPF: Australian Health Performance Framework
CIS: critical interpretive synthesis
PRISMA: Preferred Reporting Items for Systematic Reviews and Meta-Analyses
RQ: research question
