# Supplementary material for: The Unintended Consequences of Telehealth in Australia: Critical Interpretive Synthesis
Source: J Med Internet Res. 2024 Aug 27;26:e57848. doi: 10.2196/57848 (PMC11387926; doi:10.2196/57848)
Supplement: Multimedia Appendix 1 [file jmir_v26i1e57848_app1.docx]

# Multimedia Appendix 1

## Ovid MEDLINE search strategy

| 1 | Telemedicine/ or Telepathology/ or Teleradiology/ or Telerehabilitation/ or Remote Consultation/ or Distance Counseling/ or Telenursing/ |
| --- | --- |
| 2 | (telemedic* or telehealth or teleradiol* or teledermat* or telepathol* or telepsychiatr* or telerehab* or tele-medic* or tele-health or tele-radiol* or tele-dermat* or tele-pathol* or tele-psychiatr* or tele-rehab* or telecare or tele-care or telenursing or tele-nursing).tw. |
| 3 | ((remote or virtual or distance or tele or video or digital) adj2 (consult* or diagnos* or counsel* or care)).tw. |
| 4 | or/1-3 |
| 5 | ((unintended or unanticipated or unexpected or unplanned) adj2 (outcome* or consequence* or issue* or benefit* or drawback* or pitfall* or effect* or result*)).mp. |
| 6 | (evaluat* or assess*).tw. |
| 7 | (utili?ation or delivery or implementation).tw. |
| 8 | or/5-6 |
| 9 | exp australia/ or (australia* or northern territory or tasmania or new south wales or Victoria or queensland or south Australia or western australia).ti,ab. |
| 10 | and/4,8-9 |
| 11 | limit 10 to english language |

## Ovid Embase search strategy

| 1 | Telemedicine/ or Telepathology/ or Teleradiology/ or Telerehabilitation/ or Remote Consultation/ or Distance Counseling/ or Telenursing/ |
| --- | --- |
| 2 | (telemedic* or telehealth or teleradiol* or teledermat* or telepathol* or telepsychiatr* or telerehab* or tele-medic* or tele-health or tele-radiol* or tele-dermat* or tele-pathol* or tele-psychiatr* or tele-rehab* or telecare or tele-care or telenursing or tele-nursing).tw. |
| 3 | ((remote or virtual or distance or tele or video or digital) adj2 (consult* or diagnos* or counsel* or care)).tw. |
| 4 | or/1-3 |
| 5 | ((unintended or unanticipated or unexpected or unplanned) adj2 (outcome* or consequence* or issue* or benefit* or drawback* or pitfall* or effect* or result*)).mp. |
| 6 | (evaluat* or assess*).tw. |
| 7 | (utili?ation or delivery or implementation).tw. |
| 8 | or/5-6 |
| 9 | exp australia/ or (australia* or northern territory or tasmania or new south wales or Victoria or queensland or south Australia or western australia).ti,ab. |
| 10 | and/4,8-9 |
| 11 | limit 10 to english language |

## EBSCO CINAHL search strategy

| S1 | (MH "Telehealth") OR (MH "Telemedicine") OR (MH "Remote Consultation") OR (MH "Telepathology") OR (MH "Teleradiology") OR (MH "Telepsychiatry") OR (MH "Telenursing") OR (MH "Telerehabilitation") |
| --- | --- |
| S2 | TI ( telemedic* or telehealth or teleradiol* or teledermat* or telepathol* or telepsychiatr* or telerehab* or tele-medic* or tele-health or tele-radiol* or tele-dermat* or tele-pathol* or tele-psychiatr* or tele-rehab* or telecare or tele-care telenursing or tele-nursing ) OR AB ( telemedic* or telehealth or teleradiol* or teledermat* or telepathol* or telepsychiatr* or telerehab* or tele-medic* or tele-health or tele-radiol* or tele-dermat* or tele-pathol* or tele-psychiatr* or tele-rehab* or telecare or tele-care or telenursing or tele-nursing ) |
| S3 | TI ( (remote or virtual or distance or tele or video or digital) N2 (consult* or diagnos* or counsel* or care) ) OR AB ( (remote or virtual or distance or tele or video or digital) N2 (consult* or diagnos* or counsel* or care) ) |
| S4 | S1 OR S2 OR S3 |
| S5 | TX (unintended or unanticipated or unexpected or unplanned) N2 (outcome* or consequence* or issue* or benefit* or drawback* or pitfall* or effect* or result*) |
| S6 | TI (evaluat* or assess* ) OR AB ( evaluat* or assess*) |
| S7 | TI ( utili?ation or delivery or implementation ) OR AB ( utili?ation or delivery or implementation ) |
| S8 | S5 OR S6 OR S7 |
| S9 | MH "Australia+" OR TI ( australia* or australian capital territory or northern territory or tasmania or new south wales or Victoria or queensland or south Australia or western australia ) OR AB ( australia* or australian capital territory or northern territory or tasmania or new south wales or Victoria or queensland or south Australia or western australia ) |
| S10 | S4 AND S8 AND S9 |
| S11 | S10 - limit by language - english |

## Scopus search strategy

| **1** | TITLE-ABS-KEY ( ( ( telemedic* OR telehealth OR teleradiol* OR teledermat* OR telepathol* OR telepsychiatr* OR telerehab* OR tele-medic* OR tele-health OR tele-radiol* OR tele-dermat* AND tele-pathol* OR tele-psychiatr* OR tele-rehab* OR telecare OR tele-care OR telenursing OR tele-nursing ) OR ( ( remote OR virtual OR distance OR tele OR video OR digital ) W/2 ( consult* OR diagnos* OR counsel* OR care ) ) ) ) |
| --- | --- |
| **2** | ALL ( ( ( unintended OR unanticipated OR unexpected OR unplanned ) W/2 ( outcome* OR consequence* OR issue* OR benefit* OR drawback* OR pitfall* OR effect* OR result* ) ) )  ( TITLE-ABS-KEY ( evaluat* OR assess* ) OR TITLE-ABS-KEY ( implementation or utili*ation or delivery ) ) |
| **3** | TITLE-ABS-KEY ( australia* OR australian AND capital AND territory OR northern AND territory OR tasmania OR new AND south AND wales OR victoria OR queensland OR south AND australia OR western AND australia ) |
| **4 = 1 AND 2 AND 3** | ( TITLE-ABS-KEY ( ( ( telemedic* OR telehealth OR teleradiol* OR teledermat* OR telepathol* OR telepsychiatr* OR telerehab* OR tele-medic* OR tele-health OR tele-radiol* OR tele-dermat* AND tele-pathol* OR tele-psychiatr* OR tele-rehab* OR telecare OR tele-care OR telenursing OR tele-nursing ) OR ( ( remote OR virtual OR distance OR tele OR video OR digital ) W/2 ( consult* OR diagnos* OR counsel* OR care ) ) ) ) ) AND ( TITLE-ABS-KEY ( ( ( unintended OR unanticipated OR unexpected OR unplanned ) W/2 ( outcome* OR consequence* OR issue* OR benefit* OR drawback* OR pitfall* OR effect* OR result* ) ) OR ( implementation OR utili*ation OR delivery ) OR (evaluat* OR assess*) ) ) AND ( TITLE-ABS-KEY ( ( australia* OR australian AND capital AND territory OR northern AND territory OR tasmania OR new AND south AND wales OR victoria OR queensland OR south AND australia OR western AND australia ) ) |
| **5 = limit 4 to English** | ( ( TITLE-ABS-KEY ( ( ( telemedic* OR telepath OR teleradiol* OR teledermat* OR telepathol* OR telepsychiatr* OR telerehab* OR tele-medic* OR tele-health OR tele-radiol* OR tele-dermat* AND tele-pathol* OR tele-psychiatr* OR tele-rehab* OR electre OR tele-care OR teleworking OR tele-nursing ) OR ( ( remote OR virtual OR distance OR tele OR video OR digital ) W/2 ( consult* OR diagnos* OR counsel* OR care ) ) ) ) ) AND ( TITLE-ABS-KEY ( ( ( unintended OR unanticipated OR unexpected OR unplanned ) W/2 ( outcome* OR consequence* OR issue* OR benefit* OR drawback* OR pitfall* OR effect* OR result* ) ) OR ( implementation OR utili*ation OR delivery ) OR ( evaluat* OR assess* ) ) ) AND ( TITLE-ABS-KEY ( australia* OR "australian capital territory" OR "northern territory" OR tasmania OR "new south wales" OR victoria OR queensland OR "south australia" OR "western australia" ) ) ) AND ( LIMIT-TO ( LANGUAGE , "English" ) ) |
